# Oral Hygiene Practices of Hospitalized Patients in Public and Private Hospitals in Al-Ahsa, Saudi Arabia: A Cross-Sectional Study

**DOI:** 10.3390/jcm14248698

**Published:** 2025-12-09

**Authors:** Amany Osama Kassem, Muhammad Farooq Umer, Mohammad Alhussein Hamidaddin, Elwalid Fadul Nasir, Areej Jafar Alomran, Hajar Ibrahim Alsuwayi, Mohammad Abdullah AlQahtani, Nazargi Mahabob Basha, Syed Akhtar Hussain Bokhari

**Affiliations:** 1 Department of Preventive Dental Sciences, College of Dentistry, King Faisal University, Hofuf 31982, AlAhsa, Saudi Arabia; osama_amany@ymail.com (A.O.K.); mhamidaddin@kfu.edu.sa (M.A.H.); enasir@kfu.edu.sa (E.F.N.); sbokhari@kfu.edu.sa (S.A.H.B.); 2 Riyadh Second Health Cluster, Ministry of Health, Riyadh City 11176, Saudi Arabia; areej.j.alomran@gmail.com; 3 Eastern Health Cluster, Ministry of Health, Dammam City 31441, Saudi Arabia; hajar.i.alsuwayi@gmail.com; 4 Faisal Tabuk Dental, Tabuk City 71421, Saudi Arabia; qahtanimohammad95@gmail.com; 5 Department of Oral and Maxillofacial Surgery and Diagnostic Sciences, College of Dentistry, King Faisal University, Hofuf 31982, AlAhsa, Saudi Arabia; nmahabob@kfu.edu.sa

**Keywords:** oral hygiene, hospitalized patients, Saudi Arabia, hospital-acquired pneumonia, cross-sectional study, oral care practices, patient safety, healthcare providers

## Abstract

**Background/Objectives:** Oral hygiene is an essential component of overall health, but is often neglected during hospital stays, particularly among patients who rely on healthcare providers for daily care. Poor oral hygiene may lead to discomfort, infections, and complications such as hospital-acquired pneumonia. The objective of this study was to assess the oral hygiene practices of hospitalized patients in Al-Ahsa, Saudi Arabia. **Methods:** A hospital-based cross-sectional study was conducted among patients in public and private hospitals. Since no prior studies existed for this population, a pilot study with 60 participants was used to estimate the population proportion for sample size calculation. Based on the pilot findings, a proportion of 80% was assumed, with a 95% confidence level, 5% margin of error, and 80% power. Patients were recruited through non-probability convenience sampling. Data were collected via structured face-to-face interviews and analyzed using SPSS version 27. Descriptive statistics, chi-square tests, and logistic regression were applied, with significance set at 0.05. **Results:** Regular toothbrushing declined from 69.6% before admission to 29.8% during hospitalization. Only 29.8% of patients received oral hygiene supplies, and 79.2% received no assistance. In-hospital toothbrushing was significantly associated with being female (AOR = 2.52; 95% CI: 1.17–5.43), non-Saudi (AOR = 3.91; 95% CI: 1.22–12.55), and having a Bachelor’s degree or higher (AOR = 5.66; 95% CI: 1.53–20.88). **Conclusions:** Oral hygiene among hospitalized patients in Al-Ahsa was inadequate, particularly in public hospitals where essential supplies were lacking. Hospitals should adopt clear oral care policies, ensure supply availability, train staff, and integrate dental professionals to improve patient safety and prevent complications.

## 1. Introduction

Oral hygiene is a fundamental component of general health and quality of life. However, during hospitalization, it is often neglected, particularly among patients who experience physical, cognitive, or functional limitations that interfere with self-care [1,2]. In hospitalized individuals—especially those who are elderly, dependent, or medically compromised—oral hygiene frequently deteriorates due to fatigue, immobility, medication side effects, or reliance on others for assistance. Poor oral hygiene in hospital settings has been associated with numerous local and systemic complications, including plaque accumulation, gingival inflammation, oral infections, halitosis, and oral discomfort [3,4]. Neglecting oral care further increases the risk of hospital-acquired infections such as aspiration pneumonia, especially among dependent or critically ill patients [5,6]. Within 48 h of admission, dental plaque can become colonized by pathogenic microorganisms if not adequately cleaned [4]. Consequently, compromised oral health may adversely affect nutritional intake, communication, comfort, and overall well-being [3].

Hospitalization is frequently accompanied by a decline in oral hygiene behaviors, as many patients are unable to maintain routine practices such as toothbrushing and mouth rinsing [7]. This deterioration has been linked to limited access to oral care materials, dependence on nursing staff, and the absence of standardized oral hygiene protocols within hospital settings [8]. Evidence from Saudi Arabia indicates that hospitalized patients exhibit poorer oral health compared with nonhospitalized individuals, emphasizing the need to strengthen oral care programs in hospitals [9]. Inadequate staff training, heavy workload, and limited institutional focus on oral health have also been identified as major barriers to effective oral hygiene support [10].

This study is also grounded conceptually in Orem’s Self-Care Deficit Nursing Theory, which posits that individuals have a natural capacity for self-care, but hospitalization can significantly reduce this ability, making them reliant on nursing staff to meet fundamental hygiene needs. In the context of oral health, hospitalized patients—particularly those who are acutely ill, immobilized, or medicated—experience reduced self-care agency, increasing their vulnerability to oral hygiene deterioration. Orem’s model highlights that when patients cannot perform self-care independently, the responsibility shifts to healthcare providers to prevent oral complications. This theoretical perspective underscores the importance of assessing oral hygiene practices delivered by hospital staff, as inadequate support may contribute to preventable oral and systemic complications [11].

The clinical consequences of poor oral care extend beyond discomfort, as inadequate oral hygiene is a preventable factor contributing to hospital-acquired infections. Structured oral hygiene programs have been shown to reduce the incidence of non-ventilator hospital-acquired pneumonia and other complications among medical and surgical patients [12]. Nevertheless, oral care practices remain inconsistent across healthcare institutions due to insufficient training, time constraints, and the absence of standardized protocols [13]. Nurses often report a lack of institutional support and evidence-based guidelines for oral care, contributing to variability in delivery [13]. Moreover, the lack of systematic oral health education during hospital admission limits patients’ awareness of preventive hygiene practices, thereby increasing infection risk and compromising patient safety [14].

Despite global recognition of oral hygiene as an essential element of patient safety, studies exploring the oral hygiene practices of hospitalized adults in Saudi Arabia remain limited. In particular, data from the Al-Ahsa region concerning patients’ oral care behaviors and the institutional support provided are scarce. Understanding these aspects is essential, as oral hygiene forms an integral part of Saudi Vision 2030, which prioritizes preventive healthcare, patient safety, and quality of care [15].

The purpose of this study was to assess the oral hygiene practices of hospitalized patients in public and private hospitals in Al-Ahsa, Saudi Arabia. By identifying existing practices, gaps, and barriers, this research aims to provide evidence-based recommendations for developing standardized oral care protocols and institutional policies aligned with national health priorities and international patient safety goals.

## 2. Materials and Methods

### 2.1. Study Design, Setting, and Participants

This hospital-based cross-sectional analytical study was conducted in Al-Ahsa, Eastern Province, Saudi Arabia, between March 2024 and May 2025. The overall period covered all stages of the project, including ethical approvals, instrument validation, pilot testing, data collection, data analysis, and manuscript preparation. Al-Ahsa is the largest governorate in the Kingdom, encompassing both urban and rural regions and served by a well-established healthcare system. The region comprises a total of twelve hospitals, including eight public and four private institutions, all of which provide inpatient care. To ensure a balanced and representative sample, the study included four public hospitals and two private hospitals [16].

**Figure 1:**
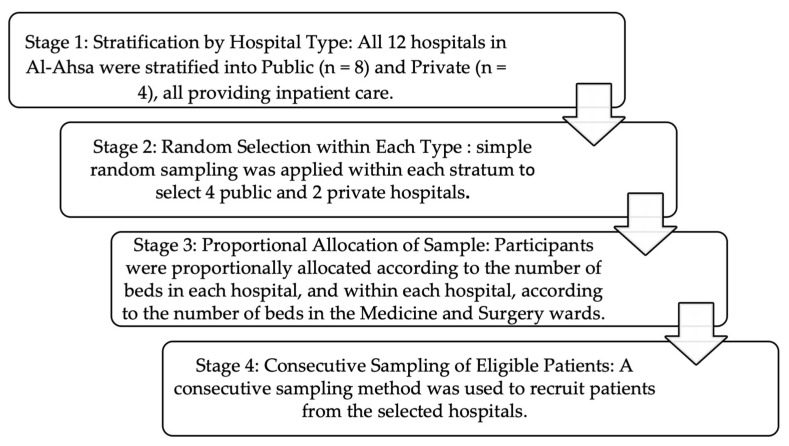
Flowchart illustrating the sampling procedure for hospitals and patients in Al-Ahsa, Saudi Arabia.

A multistage stratified sampling design was used at the institutional level, followed by consecutive sampling at the individual level (Figure 1).

The study population included adult hospitalized patients admitted to internal medicine and surgical wards. Inclusion criteria were: being 18 years of age or older, hospitalized for a minimum of three consecutive days in alignment with the Saudi Ministry of Health’s definition of long-term hospitalization to ensure a valid and meaningful assessment of oral-hygiene practices, being conscious at the time of data collection, and able to communicate in either Arabic or English. Patients with cognitive impairments due to psychiatric conditions such as schizophrenia, Alzheimer’s disease, or severe bipolar disorder were excluded.

Since no prior studies had been conducted on oral-hygiene practices of hospitalized patients in the Al-Ahsa region, a pilot study involving 60 participants was conducted to assess feasibility and estimate the population proportion for sample-size calculation [17,18]. Based on the pilot study findings, an anticipated population proportion of 80% was used, with a 95% confidence level, 5% margin of error, and 80% statistical power. The sample size was calculated using a standard formula for estimating proportions in a finite population. The following formula was applied:

$$n=\frac{N\cdot \left(\frac{Z^{2}\cdot p\cdot \left(1-p\right)}{{MOE}^{2}}\right)}{\left(\frac{Z^{2}\cdot p\cdot \left(1-p\right)}{{MOE}^{2}}\right)+N-1}$$

Accordingly, the required sample size for hospitalized patients was approximately 171, drawn from a population of 566. After proportional allocation across the selected hospitals, consecutive sampling was used only at the final stage to recruit eligible patients within each Medicine and Surgery ward until the allocated sample size was achieved.

### 2.2. Study Tool

Data collection was conducted using a structured, validated, and interviewer-administered questionnaire. The original version was developed in English, translated into Arabic, and then back-translated to ensure conceptual and linguistic equivalence. The questionnaire underwent a multi-step validation process prior to data collection. First, face validation was conducted by two oral public health specialists and three hospital-based nurses to ensure clarity, relevance, and appropriateness of the items for hospitalized patients. Content validation was performed using an expert panel of five dental public health academics who assessed each item for relevance, simplicity, and necessity. The instrument was subsequently pilot-tested among 20 hospitalized patients not included in the main study sample to evaluate comprehension, clarity, and response consistency. Minor modifications were made to improve wording and reduce ambiguity. Test–retest reliability demonstrated acceptable stability, with Cohen’s κ values ranging from 0.65 to 0.95. Internal-consistency reliability was confirmed with a Cronbach’s α of 0.74.

The final questionnaire comprised 49 closed-ended items organized into four domains: (1) sociodemographic and hospitalization-related characteristics; (2) oral-hygiene practices before and during hospitalization; (3) perceived barriers and oral-health education; and (4) oral-health problems experienced during hospitalization. To ensure contextual relevance and methodological rigor, the instrument was adapted from previously validated tools used in similar hospital-based oral-health studies that explored patients’ oral-hygiene status, barriers to care, and the impact of hospitalization on oral health [7,19,20,21].

### 2.3. Study Variables

The primary outcome variable was oral-hygiene practice during hospitalization, which was examined in relation to a set of independent variables encompassing various sociodemographic characteristics, including age, gender, nationality, marital status, education level, employment status, place of residence, and monthly household income [22,23,24], as well as hospital-related factors such as hospital type (public or private), ward of admission (medical or surgical), length of hospital stay, presence of systemic health conditions, and previous history of hospitalization [23,24]. Variable categorization was guided by established international frameworks, including the International Standard Classification of Education (ISCED-11) and the Saudi Ministry of Health guidelines for hospital classification [24].

### 2.4. Statistical Analysis

Data were analyzed using IBM SPSS Statistics for Windows, Version 27.0 (IBM Corp., Armonk, NY, USA). Descriptive statistics, including means, standard deviations, frequencies, and percentages, were computed to summarize participants’ characteristics and key study variables. Associations between categorical variables were examined using the Chi-square test or Fisher’s exact test, depending on the data distribution. To explore the relationship between selected variables, multivariable logistic regression analysis was performed, and results were expressed as adjusted odds ratios (AORs) with corresponding 95% confidence intervals (CIs). A *p*-value of ≤0.05 was considered statistically significant.

## 3. Results

The findings describe the characteristics of the study participants and their oral hygiene practices in relation to hospital type and care conditions.

### 3.1. Patient Characteristics and Hospitalization Profile

The mean age of the hospitalized patients was 46.9 ± 18.7 years (95% CI: 44.1–49.7). Most were male (58.5%) and Saudi nationals (82.5%). Nearly half were unemployed (45.0%), and the most frequent monthly income was <5000 SR (39.2%). Slightly more patients were admitted to private hospitals (53.2%) than to public hospitals (46.8%). The majority stayed three to seven days (59.1%), and acute illness was the most common cause of admission (60.8%). One-third reported no systemic diseases (33.3%), and most had been hospitalized previously (74.3%), with a median of three prior admissions (Table 1 and Table 2).

### 3.2. Oral Hygiene Practices Before and During Hospitalization

Before hospitalization, one-third of patients brushed twice daily (32.2%) and (29.2%) once daily, while (9.9%) did not brush at all. Mouthwash (17.5%) and floss (11.1%) use were low, and miswak use was reported only in public hospitals, showing a significant difference between hospital types (*p* = 0.004). During hospitalization, oral hygiene practices declined markedly, with only 36.3% brushing their teeth, and most began to brush after the first day. The reasons for this delay were assessed among the subgroup of patients who brushed during hospitalization but not on the first day *(n* = 41), revealing that lack of toothbrush availability was the main barrier in public hospitals, whereas medical conditions were the predominant barrier in private facilities (*p* < 0.001). More than half of the patients (56.1%) were unable to clean independently, and 79.2% reported receiving no assistance. Overall, brushing frequency, mouthwash use, and miswak use dropped significantly during hospitalization, with private hospitals showing better patient self-care ability than public hospitals (*p* = 0.010) (Table 3).

### 3.3. Availability of Oral Hygiene Supplies and Oral Care Services

A minority of patients (29.8%) reported receiving oral hygiene supplies from the hospital, while the majority did not (67.8%). Among those who did not receive supplies, 31.0% brought their own. There was strong agreement among patients that hospitals should provide oral hygiene supplies, with 77.8% strongly agreeing and 15.8% agreeing. No patients reported receiving any information about oral care during their hospital stay. A small percentage of patients (9.4%) received an oral health assessment, conducted exclusively by doctors. Among these assessments, the most common time for the examination was before surgery or other major procedures (50.0%) (Table 4).

### 3.4. Satisfaction with Care and Oral Health Issues

Among the patients who received an oral health examination, the majority were neutral about it (75.0%), while smaller proportions were very satisfied (6.3%), satisfied (12.5%), or very dissatisfied (6.3%). A minority of all patients (17.5%) experienced oral health issues during their hospital stay. The most common issues were dry mouth (50.0%), mouth sores (30.0%), and difficulty swallowing (20.0%). Among those who experienced oral health issues, the most common response regarding how these issues were addressed was that no concrete efforts were made (60.0%), followed by management with basic intervention (30.0%) (Table 5).

### 3.5. Comparison of Oral Hygiene Practices and Support Services Between Public and Private Hospitals

Approximately one-third of patients (36.3%) had their teeth cleaned during hospitalization, with no significant difference between hospital types (*p* = 0.52). Independent oral hygiene was significantly higher in private hospitals (53.8%) than in public ones (32.5%) (OR = 0.41, 95% CI: 0.22–0.77, *p* = 0.005). Assistance with oral care was limited (15.0%) and similar across hospitals (*p* = 0.310). Regular toothbrushing during hospitalization was uncommon (29.8%), more frequent in private hospitals (36.3%) than in public hospitals (22.5%) (*p* = 0.070). The provision of oral hygiene supplies was significantly greater in private hospitals (57.1%) compared to public hospitals (3.8%) (*p* < 0.001). Only 9.4% of patients received an oral health assessment, slightly higher in private hospitals (13.2%) than in public ones (5.0%) (*p* = 0.067) (Table 6).

### 3.6. Predictors of Toothbrushing Behavior Among Hospitalized Patients

Multivariable logistic regression identified key predictors of toothbrushing during hospitalization. Female patients were more than twice as likely to brush compared with males (AOR = 2.52, 95% CI: 1.17–5.43, *p* = 0.018), and non-Saudis were nearly four times more likely to brush than Saudis (AOR = 3.91, 95% CI: 1.22–12.55, *p* = 0.022). Higher education also showed a significant positive association, with patients holding a bachelor’s degree or above being over five times more likely to brush (AOR = 5.66, 95% CI: 1.53–20.88, *p* = 0.009). Age, hospital type, department, and length of stay were not significantly related to toothbrushing behavior (Table 7).

## 4. Discussion

This study provides valuable insights into the oral hygiene practices of hospitalized patients in Al-Ahsa, Saudi Arabia, highlighting several deficiencies that extend beyond the mere availability of supplies. Oral care was not systematically incorporated into routine hospital practices, as evidenced by infrequent toothbrushing, limited use of oral hygiene aids, minimal assistance provided to dependent patients, and the lack of structured oral health education or assessment. These behavioral and institutional gaps may contribute to an increased risk of preventable oral and systemic complications, such as aspiration pneumonia and other hospital-acquired infections. The findings underscore the need to recognize oral hygiene as a critical element of patient safety and infection-control protocols within hospital settings.

Overall, oral hygiene practices during hospitalization were inadequate. Most patients did not maintain regular brushing routines, and the use of mouthwash, dental floss, or miswak was uncommon. This decline was compounded by the limited availability of basic oral care materials, which were significantly more available in private than in public hospitals. Patients in facilities with better supply provision demonstrated higher brushing frequency, indicating that access to resources and administrative prioritization strongly influence patient behavior. More than half of the hospitalized patients were unable to clean their teeth independently, and only a small proportion received assistance from nurses. The absence of oral health education and structured oral hygiene assessments further highlights systemic neglect, underscoring the need for institutional policies to ensure consistent supply provision, staff participation, and patient support.

These results align with international evidence showing that inadequate provision of oral care supplies remains a challenge across healthcare systems. Studies from Iran and Germany reported similar shortcomings, where essential oral hygiene items were inconsistently distributed among hospitalized patients [8,25]. In several Asian contexts, patients often rely on family members to provide oral hygiene products [26,27]. In contrast, Italian hospitals achieved substantially higher rates of toothbrush use (65–100%) through standardized oral care programs and reliable supply distribution [7]. These comparisons emphasize the influence of institutional policy, leadership engagement, and structured oral care protocols on patient outcomes. The absence of comparable frameworks in Saudi hospitals, particularly within the public sector, underscores the need for policy-driven interventions to ensure equitable access to basic oral hygiene resources.

Equally concerning was the minimal nursing assistance provided to hospitalized patients who were unable to perform oral care independently. Only a small proportion of dependent patients reported receiving help from nurses, a figure markedly lower than that documented in Eritrea [8] and far below rates in health systems where oral care is embedded in nursing competencies. Contributing factors may include excessive workload, staffing shortages, and the absence of clear institutional guidelines emphasizing oral hygiene as a component of nursing care. Previous research has shown that when oral care is excluded from nursing documentation or daily checklists, its delivery becomes inconsistent and dependent on individual initiative and prior training [9,28]. Within Saudi healthcare institutions, high patient loads and limited time allocation likely exacerbate this gap, reinforcing the need for standardized protocols, staff accountability, and continuous monitoring mechanisms.

Another key finding was the complete absence of structured oral health education and routine oral hygiene assessment. None of the participants reported receiving guidance on maintaining oral hygiene while hospitalized, and only a small fraction underwent oral examinations, usually by physicians before surgical procedures. Hospitalization often disrupts daily self-care routines, particularly among patients with restricted mobility or medical contraindications, leaving them uncertain about appropriate oral hygiene practices. Evidence consistently demonstrates that oral health education enhances adherence to oral care and reduces complications such as aspiration pneumonia [12,14]. Programs such as Mouth Care Matters in the United Kingdom have shown that combining staff training, standardized protocols, and oral health education can significantly improve oral hygiene outcomes and patient safety [6]. Similarly, nurse-led oral care initiatives in Japan and Australia have reduced hospital-acquired pneumonia and improved overall patient well-being [29,30]. The lack of comparable programs in Saudi Arabia represents a key opportunity for capacity building and integration of oral health promotion within hospital systems.

Sociodemographic variations observed in this study further illustrate the influence of cultural, educational, and behavioral factors on oral hygiene practices among hospitalized patients. Female patients, non-Saudi nationals, and those with higher educational attainment were more likely to maintain oral hygiene during hospitalization than their male, Saudi, and less-educated counterparts. These findings are consistent with previous studies showing that women and individuals with higher education levels exhibit greater health awareness and preventive care behaviors [20,31]. Similar evidence from Middle Eastern contexts indicates that health literacy and prior exposure to preventive dental care predict better oral hygiene behaviors [32,33]. Accordingly, oral health promotion strategies should be culturally and linguistically tailored to ensure accessibility for patients with diverse backgrounds and varying literacy levels.

The associations observed with gender, nationality, and educational level may be explained by underlying sociocultural and behavioral mechanisms. Women have been shown to exhibit greater concern for personal hygiene and may be more likely to report or request assistance with oral hygiene, which could explain gender differences observed in this study. Nationality-related disparities may reflect cultural norms surrounding hygiene behaviors, patient–provider interactions, and expectations regarding the role of nursing staff in personal care. Educational level is strongly linked to health literacy, which influences awareness of oral hygiene importance, ability to communicate needs, and familiarity with preventive practices. These sociocultural and literacy-related factors likely contribute to variations in oral hygiene practices among hospitalized patients and warrant consideration when designing hospital-based oral care protocols.

The implications of these findings extend beyond clinical care to institutional and policy domains. Hospitals should ensure the consistent availability of basic oral hygiene supplies as part of standard admission procedures and incorporate oral care into nursing documentation, supervision, and performance evaluation. Continuing professional development programs should emphasize the infection-control benefits of oral hygiene and provide practical training for assisting dependent patients. Structured oral health education, introduced at admission and reinforced throughout hospitalization, should be an integral component of multidisciplinary care.

The variation in oral healthcare practices observed between public and private hospitals was minimal, indicating that both sectors face comparable challenges in implementing effective oral hygiene protocols. Although private hospitals demonstrated slightly better access to oral hygiene supplies and marginally higher self-care rates, these differences were not statistically significant overall. This suggests that the gaps in oral care during hospitalization are systemic rather than institution-specific. Addressing these issues, therefore, requires broader strategies—such as ensuring the regular supply of oral hygiene materials, incorporating oral care into nursing routines, and providing continuous staff training across all hospital types—to promote consistent and equitable standards of patient care.

Future research should focus on developing and evaluating targeted oral care interventions within hospital settings, including nurse-led protocols, staff training modules, and patient education programs. Longitudinal and interventional studies are needed to assess the effectiveness of these measures in reducing oral health complications, improving patient satisfaction, and integrating oral hygiene into national hospital accreditation and quality standards. Although this study provides valuable insights, its findings may not be directly applicable to other hospital departments beyond Medicine and Surgery wards, where clinical priorities and patient characteristics differ.

The use of consecutive sampling in this study, although practical and commonly used in hospital-based research, introduces potential selection bias. Because participants were recruited based on their sequence of admission and availability during the data collection period, certain groups may have been overrepresented—for example, patients admitted on specific days, at certain times, or in particular wards with higher patient turnover. This limits the generalizability of the findings to the broader hospitalized population, including patients from specialized units or those admitted during off-peak periods. Consecutive sampling was selected due to its feasibility and suitability in real-time clinical settings. Another important limitation is the study’s reliance on self-reported oral hygiene practices and perceived oral health conditions without accompanying clinical dental assessments. Self-reported data are prone to recall bias, social desirability bias, and subjective interpretation, which may lead participants to underreport or overreport their actual oral hygiene status. The absence of an objective clinical examination limits the ability to validate patient-reported findings or detect subclinical oral health problems such as early carious lesions, periodontal inflammation, or plaque accumulation. This methodological constraint was largely due to restrictions within hospital wards, where routine dental examination equipment and infection control protocols were not available. Future studies should incorporate standardized clinical dental assessments or hybrid approaches combining self-report with clinical indices to enhance the accuracy and robustness of oral health evaluation. A further limitation of this study is the lack of information on important clinical and patient-related variables such as functional status, cognitive status, diet type, and underlying comorbidities. These factors have a substantial influence on a patient’s ability to perform oral hygiene independently and their reliance on nursing assistance. The omission of these data was primarily due to variability in documentation across the participating wards and the unavailability of standardized assessments within general medical and surgical units.

## 5. Conclusions

Oral hygiene practices among hospitalized patients in Al-Ahsa were generally inadequate, reflecting both behavioral and institutional shortcomings. Although private hospitals showed slightly better performance in supply provision and independent oral care, the overall quality of oral hygiene practices remained low across both sectors. The absence of structured oral health education, limited staff assistance, and poor supply availability highlight the need for comprehensive hospital-based oral care policies. Incorporating oral hygiene into standard nursing care plans, providing continuous staff training, involving dental professionals in inpatient care, and ensuring consistent access to oral hygiene materials are essential steps toward improving patient safety and quality of care. These measures would help reduce preventable oral and systemic complications and promote equitable oral health standards for all hospitalized patients.

These findings have clear implications for ongoing national reforms under Saudi Arabia’s Vision 2030 and its Health Sector Transformation Program (HSTP). Improving oral hygiene practices in hospitals aligns directly with Vision 2030 priorities that emphasize preventive care, reduction in hospital-acquired infections, and enhancement of patient safety standards. Integrating structured oral hygiene protocols, staff training, and standardized assessment tools into routine inpatient care can strengthen care quality, reduce complications such as non–ventilator-associated pneumonia, and optimize resource use. As the health system shifts toward value-based, patient-centered care, strengthening oral hygiene support in hospitals represents a practical and cost-effective strategy to advance the quality and safety benchmarks outlined in Vision 2030.

## Figures and Tables

**Table 1:** Sociodemographic characteristics of hospitalized patients in Al-Ahsa, Saudi Arabia (*n* = 171).

Socio-Demographic Data	No	%
**Age in years**		
18–29	37	(21.5)
30–39	28	(16.4)
40–49	34	(19.9)
50–59	23	(13.5)
60–69	29	(17.0)
70+	20	(11.7)
Mean ± SD (95% CI)	46.9 ± 18.7 (44.1–49.7)	
**Gender**		
Male	100	(58.5)
Female	71	(41.5)
**Nationality**		
Saudi	141	(82.5)
Non-Saudi	30	(17.5)
**Marital status**		
Single	41	(24.0)
Married	109	(63.7)
Divorced/widow	21	(12.3)
**Place of Residency**		
Al-Hofuf	68	(39.8)
Al-Mubarraz	42	(24.6)
Al-Oyun	5	(2.9)
Village	56	(32.7)
**Educational level**		
Non-educated	23	(13.5)
Below secondary education	49	(28.7)
Secondary/diploma	57	(33.3)
Bachelor degree/above	42	(24.6)
**Employment**		
Unemployed	77	(45.0)
Governmental employee	14	(8.2)
Self-employed	5	(2.9)
Private sector employee	48	(28.1)
Retired	27	(15.8)
**Monthly income**		
<5000 SR	67	(39.2)
5000–9999 SR	61	(35.7)
10,000–15,000 SR	34	(19.9)
>15,000 SR	9	(5.3)

Bold text indicates the questionnaire items (questions), while unbolded text indicates the response choices.

**Table 2:** Medical and Hospitalization data of Hospitalized Patients in Al-Ahsa, Saudi Arabia *(n* = 171).

Medical and Hospitalization	No	%
**Type of hospital you are currently admitted to**		
Public hospital	80	(46.8)
Private hospital	91	(53.2)
**Ward you are currently admitted to**		
Medicine	88	(51.5)
Surgery	83	(48.5)
**Length of Current Hospital Stay (in days)**		
3–7 days	101	(59.1)
7–14 days	45	(26.3)
>14 days	25	(14.6)
**The cause of your hospitalization**		
Acute illness	104	(60.8)
Chronic condition	67	(39.2)
**Do you have any systemic diseases?**		
No systemic diseases	57	(33.3)
One systemic disease	63	(36.8)
More than one systemic disease	51	(29.8)
**How are you covering your medical expenses as a hospitalized patient?**		
Entitled to free healthcare (complete coverage by government)	72	(42.1)
Insurance	98	(57.3)
Out-of-pocket expenses	1	(0.6)
**Have you ever been hospitalized before?**		
Yes	127	(74.3)
No	44	(25.7)
**When was your last hospitalization?**		
Within the last 3 months	49	(38.6)
Within the last 6 months	21	(16.5)
Within the last 1 year	11	(8.7)
Over 1 year ago	46	(36.2)
**How many times of previous hospitalizations**		
Median (Range)	3 (1–67)	

Bold text indicates the questionnaire items (questions), while unbolded text indicates the response choices.

**Table 3:** Oral Hygiene Practices of Hospitalized Patients Before and During Hospitalization in Public and Private Hospitals in Al-Ahsa, Saudi Arabia (*n* = 171).

Items	All No (%)	Public No (%)	Private No (%)	*p*-Value
**Before Hospitalization**				
**How often do you brush your teeth?**				
I don’t brush my teeth	17 (9.9)	9 (11.2)	8 (8.8)	0.940
Once a week	12 (7.0)	5 (6.2)	7 (7.7)	
Twice a week	7 (4.1)	4 (5.0)	3 (3.3)	
Every other day (alternating days)	16 (9.4)	7 (8.8)	9 (9.9)	
Once a day	50 (29.2)	21 (26.2)	29 (31.9)	
Twice a day	55 (32.2)	26 (32.6)	29 (31.9)	
More than twice a day	14 (8.2)	8 (10.0)	6 (6.6)	
**Use of mouthwash**				0.700
Yes	30 (17.5)	15 (18.8)	15 (16.5)	
No	141 (82.5)	65 (81.2)	76 (83.5)	
**Use of dental floss**				0.360
Yes	19 (11.1)	7 (8.8)	12 (13.2)	
No	152 (88.9)	73 (91.2)	79 (86.8)	
**Use of miswak**				0.004 *
Yes	7 (4.1)	7 (8.8)	0 (0.0)	
No	164 (95.9)	73 (91.2)	91 (100.0)	
**During Hospitalization**				
**Have your teeth been cleaned since admission?**				0.520
Yes	62 (36.3)	27 (33.8)	35 (38.5)	
No	109 (63.7)	53 (66.2)	56 (61.5)	
**When were your teeth first cleaned after admission?**				0.350
From the first day	21 (33.9)	6 (22.2)	15 (42.9)	
Second day	15 (24.2)	8 (29.6)	7 (20.0)	
Third day or later	26 (41.9)	13 (48.1)	13 (37.1)	
**Reasons teeth not cleaned from first day a** (*n* = 41)				<0.001 *
No toothbrush available	20 (48.8)	17 (81.0)	3 (15.0)	
No oral hygiene instructions	5 (12.2)	4 (19.0)	1 (5.0)	
Medical condition prevented brushing	16 (39.0)	0 (0.0)	16 (80.0)	
**Ability to clean teeth independently**				0.010 *
Yes	75 (43.9)	26 (32.5)	49 (53.8)	
No	96 (56.1)	54 (67.5)	42 (46.2)	
**If unable, who assists?**				0.160 ^
Nurse	3 (3.1)	2 (3.7)	1 (2.4)	
Family member	17 (17.7)	6 (11.1)	11 (26.2)	
No assistance	76 (79.2)	45 (85.2)	30 (71.4)	
**How often do you brush during stay?**				0.310 ^
I don’t brush my teeth	115 (67.3)	58 (72.5)	57 (62.6)	
Once a week	1 (0.6)	1 (1.3)	0 (0.0)	
Twice a week	1 (0.6)	1 (1.3)	0 (0.0)	
Every other day	3 (1.8)	2 (2.5)	1 (1.1)	
Once a day	26 (15.2)	11 (13.8)	15 (16.5)	
Twice a day	22 (12.9)	6 (7.5)	16 (17.6)	
More than twice a day	3 (1.8)	1 (1.3)	2 (2.2)	
**Use of mouthwash**				0.250 ^
Yes	4 (2.3)	3 (3.8)	1 (1.1)	
No	167 (97.7)	77 (96.3)	90 (98.9)	
**Use of dental floss**				0.350 ^
Yes	1 (0.6)	0 (0.0)	1 (1.1)	
No	170 (99.4)	80 (100.0)	90 (98.9)	
**Use of miswak**				0.550 ^
Yes	5 (2.9)	3 (3.8)	2 (2.2)	
No	166 (97.1)	77 (96.3)	89 (97.8)	

Bold text indicates the questionnaire items (questions), while unbolded text indicates the response choices. ^: Exact probability test, * *p* ≤ 0.05 (significant).

**Table 4:** Availability of hospital-provided oral-hygiene supplies, patient attitudes, and oral-care services during hospitalization (*n* = 171).

Service	All No (%)	Public No (%)	Private No (%)	*p*-Value
**Did the hospital provide you with oral hygiene supplies** **(e.g., toothbrush, toothpaste, mouthwash) during your stay?**				
Yes	51(29.8)	2 (2.5)	49 (53.8)	
No	116(67.8)	77 (96.3)	39 (42.9)	<0.001 *
Upon request	4 (2.3)	1 (1.3)	3 (3.3)	
**If not, did you bring your oral hygiene supplies?**				
Yes	36 (31.0)	26 (32.5)	10 (25.6)	0.370 ^
No	80 (69.0)	51 (67.5)	29 (74.4)	
**Do you think hospitals should provide oral hygiene supplies during patient stays?**				
Strongly disagree	3 (1.8)	0 (0.0)	3 (3.3)	
Neutral	8 (4.7)	1 (1.3)	7 (7.7)	0.060
Agree	27 (15.8)	15 (18.8)	12 (13.2)	
Strongly agree	133(77.8)	64 (80.0)	69 (75.8)	
**Did you receive any information about oral care during your hospital stay?**				
No	171(100)	80 (100.0)	91(100.0)	-
**Did you receive an oral health assessment during your hospital stay?**				
Yes	16(9.4)	4 (5.0)	12 (13.2)	0.070 ^
No	155(90.6)	76 (95.0)	79 (86.8)	
**Who examined your mouth?**				
Doctor	16 (100)	4 (25.0)	12 (75.0)	-
**When was the examination conducted?**				
Upon admission	3 (18.8)	0 (0.0)	3 (25.0)	
During hospital stay	4 (25.0)	1(6.3)	3 (18.8)	0.660 ^
Before surgery or other major procedures	8 (50.0)	3 (18.8)	5 (31.3)	
Based on your request	2 (12.5)	0 (0.0)	2 (12.5)	

Bold text indicates the questionnaire items (questions), while unbolded text indicates the response choices. ^: Exact probability test, * *p* ≤ 0.05 (significant).

**Table 5:** Patient Satisfaction with Oral Health Examination and Experienced Oral Health Issues During Hospital Stay in Public and Private Hospitals in Al-Ahsa, Saudi Arabia (*n* = 171).

Items	All No (%)	Public No (%)	Private No (%)	*p*-Value
**How satisfied were you with the oral health examination you received?**				
Very satisfied	1(6.3)	0 (0.0)	1 (12.5)	
Satisfied	2 (12.5)	0 (0.0)	2 (25.0)	0.760
Neutral	12(75.0)	4 (100.0)	8 (62.5)	
Very dissatisfied	1 (6.3)	0 (0.0)	1 (12.5)	
**Did you experience any oral health issues during this hospital stay?**				
Yes	30(17.5)	14 (17.5)	16 (17.6)	0.990
No	141(82.5)	66 (82.5)	75 (82.4)	
**What are the oral health issues that you faced**				
Dry mouth	15 (50.0)	6 (42.9)	9 (56.3)	
Mouth sores	9 (30.0)	6 (42.9)	3 (18.8)	
Difficulty swallowing	6 (20.0)	5 (35.7)	1 (6.3)	
Gum swelling or inflammation	5 (16.7)	3 (21.4)	2 (12.5)	0.040 *
Bleeding gums	4 (13.3)	1 (7.1)	3 (18.8)	
Tooth pain	3 (10.0)	2 (14.3)	1 (6.3)	
Oral infections (e.g., thrush)	1 (3.3)	1 (7.1)	0 (0.0)	
Fall down of the bridge	1 (3.3)	0 (0.0)	1 (6.3)	
**How were these issues addressed or treated by the hospital staff?**				
Managed with basic intervention by the physician or medical staff	9 (30.0)	3 (21.4)	6 (37.5)	
No concrete efforts were made to address the problem.	18 (60.0)	10 (71.4)	8 (50.0)	0.490
Only an examination or consultation was performed by the physician or medical staff.	3 (10.0)	1 (7.1)	2 (12.5)	

Bold text indicates the questionnaire items (questions), while unbolded text indicates the response choices. * *p* ≤ 0.05 (significant).

**Table 6:** Comparative Analysis of Oral Hygiene Practices and Support Services During Hospitalization in Public and Private Hospitals in Al-Ahsa, Saudi Arabia (*n* = 171).

Variable	All No (%)	Public No (%)	Private No (%)	Odds Ratio (95% CI)	*p*-Value
**Have your teeth been cleaned since admission?**					
Yes	62 (36.3)	27 (33.8)	35 (38.5)	0.815 (0.435–1.526)	
No	109(63.7)	53 (66.2)	56 (61.5)		0.520
**Can you clean your teeth by yourself during hospital stay?**					
Yes	75 (43.9)	26 (32.5)	49 (53.8)	0.413 (0.221–0.770)	0.005 *
No	96 (56.1)	54 (67.5)	42 (46.2)		
**Assistance with Oral Hygiene**					
Received assistance	3 (15.0)	2 (25.0)	1 (8.3)	3.67 (0.27–49.29)	0.310
Did not receive assistance	17 (85.0)	6 (75.0)	11 (91.7)		
**Tooth brushing**					
Regular brushing	51 (29.8)	18 (22.5)	33 (36.3)	1.90 (0.94–3.86)	0.070
Infrequent/Never	120 (70.2)	62 (77.5)	58 (63.7)		
**Do you use mouthwash?**					
Yes	4 (2.3)	3 (3.8)	1 (1.1)		
No	167 (97.7)	77 (96.3)	90 (98.9)	3.506 (0.357–34.403)	0.250
**Do you use dental floss?**					
Yes	1 (0.6)	0 (0.0)	1 (1.1)	**	0.350
No	170 (99.4)	80 (100.0)	90 (98.9)		
**Do you use miswak?**					
Yes	5 (2.9)	3 (3.8)	2 (2.2)	1.734 (0.282–10.647)	0.550
No	166 (97.1)	77 (96.3)	89 (97.8)		
**Hospital provided oral hygiene supplies?**					
Yes	55 (32.2)	3 (3.8)	52 (57.1)	0.029 (0.009–0.100)	<0.001 ^
No	116 (67.8)	77 (96.3)	39 (42.9)		
**If not, did you bring your supplies?**					
Yes	36 (31.0)	26 (33.8)	10 (25.6)	1.478 (0.626–3.494)	0.372
No	80 (69.0)	51 (66.2)	29 (74.4)		
**Received oral health assessment during stay?**					
Yes	16 (9.4)	4 (5.0)	12 (13.2)	0.346 (0.107–1.122)	0.067
No	155 (90.6)	76 (95.0)	79 (86.8)		

Bold text indicates the questionnaire items (questions), while unbolded text indicates the response choices. OR: Odds Ratio, CI: Confidence Interval, ^: Exact probability test, * *p* ≤ 0.05 (significant), ** CI not calculated due to zero cell value.

**Table 7:** Multivariable logistic regression analysis of factors associated with tooth-brushing among hospitalized patients (*n* = 171).

Predictor	B (SE)	AOR	95% CI	*p*-Value
**Age (years)**	0.012 (0.010)	1.01	(0.99–1.03)	0.230
**Gender**				
Male (Ref.)		1		
Female	0.923 (0.392)	2.52	(1.17–5.43)	0.018 *
**Nationality**				
Saudi (Ref.)		1		
Non-Saudi	1.363 (0.595)	3.91	(1.22–12.55)	0.022 *
**Educational level**				
Non-educated (Ref.)		1		
Below secondary education	0.340 (0.555)	1.41	(0.47–4.17)	0.540
Secondary/Diploma	0.747 (0.563)	2.11	(0.70–6.37)	0.185
Bachelor’s degree/above	1.733 (0.666)	5.66	(1.53–20.88)	0.009 *
**Hospital type**				
Public (Ref.)		1		
Private	0.157 (0.358)	1.17	(0.58–2.36)	0.661
**Department**				
Medicine (Ref.)		1		
Surgery	0.068 (0.367)	1.07	(0.52–2.20)	0.854
**Length of stay (days)**	–0.015 (0.019)	0.99	(0.95–1.02)	0.427

Bold text indicates the questionnaire items (questions), while unbolded text indicates the response choices. AOR: Adjusted Odds Ratio, CI: Confidence Interval, * *p* ≤ 0.05 (significant).

## Data Availability

Data are contained within the article.

## Abbreviations

The following abbreviations are used in this manuscript:

n	Required sample size
N	Total population size
Z	Z-value corresponding to the desired confidence level (1.96 for 95%)
p	Estimated population proportion
MOE	Margin of error
